# Oncologic outcomes of segmentectomy versus lobectomy for clinical T1c radiological pure-solid non-small-cell lung cancer

**DOI:** 10.1093/icvts/ivad152

**Published:** 2023-09-21

**Authors:** Aritoshi Hattori, Kazuya Takamochi, Takeshi Matsunaga, Mariko Fukui, Kenji Suzuki

**Affiliations:** Department of General Thoracic Surgery, Juntendo University School of Medicine, Tokyo, Japan; Department of General Thoracic Surgery, Juntendo University School of Medicine, Tokyo, Japan; Department of General Thoracic Surgery, Juntendo University School of Medicine, Tokyo, Japan; Department of General Thoracic Surgery, Juntendo University School of Medicine, Tokyo, Japan; Department of General Thoracic Surgery, Juntendo University School of Medicine, Tokyo, Japan

**Keywords:** Lung cancer, T1c, Lobectomy, Segmentectomy, Pure-solid appearance

## Abstract

**OBJECTIVES:**

We aimed to compare the outcomes of segmentectomy with those of lobectomy in T1c (>2–3 cm) radiological pure-solid non-small-cell lung cancer detected on thin-section computed tomography.

**METHODS:**

This retrospective review compared the survival outcomes, causes of death and recurrence patterns between the segmentectomy and lobectomy in patients with c-T1cN0M0 radiological pure-solid non-small-cell lung cancer. Multivariable analysis was performed to control for confounders of survival. The overall survival (OS) and recurrence-free survival were analysed using the Kaplan–Meier method. Differences in cumulative incidence of recurrence between groups were assessed using the methods of Gray.

**RESULTS:**

Of the 426 patients, lobectomy was performed in 381 patients and segmentectomy in 45 patients. Nodal metastasis was noted in 104 (24.4%) patients. Multivariable analysis revealed that lobectomy was an independent prognosticator of better OS (hazard ratio 0.596, 95% confidence interval 0.366–0.969; *P* = 0.037). Lobectomy arm showed favourable 5-year OS and recurrence-free survival (OS: 72.9% vs 59.7%, log-rank test *P* = 0.007; recurrence-free survival: 64.4% vs 48.7%, *P* = 0.034) (median follow-up: 53 months). Approximately 14% of the patients in the lobectomy group and 27% in the segmentectomy group died of lung cancer. Furthermore, 5-year cumulative incidence of loco-regional recurrence rate was significantly higher in the segmentectomy group (35.5% vs 15.8%, *P* < 0.001).

**CONCLUSIONS:**

In T1c radiological pure-solid non-small-cell lung cancer, segmentectomy was significantly associated with worse survival and insufficient loco-regional cancer control. Lobectomy remains the standard surgical treatment; meanwhile, segmentectomy should be applied with great caution.

**Figure:**
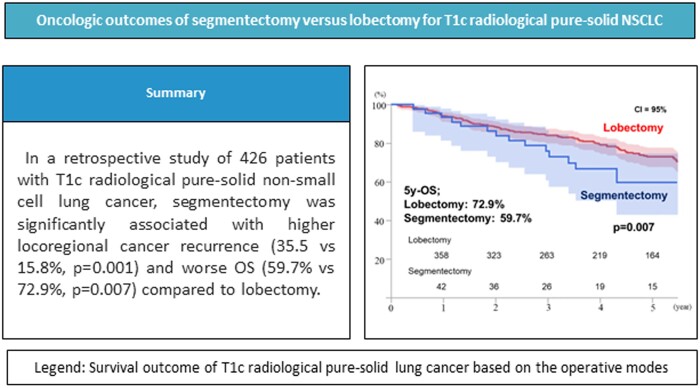

## INTRODUCTION

Recent phase III randomized trials have demonstrated the favourable survival outcomes of segmentectomy for early-stage non-small-cell lung cancer (NSCLC) [1–3]. These results indicate that segmentectomy is an alternative surgical method to lobectomy for patients with small-sized peripheral c-stage IA NSCLC [1, 2]. Based on these pivotal results, the next clinical concern is whether the surgical indication for anatomical segmentectomy can be expanded to patients with clinical-T1c NSCLC. However, lobectomy with mediastinal nodal dissection remains the primary surgical strategy for NSCLC of >2 cm in size from the point of loco-regional cancer control [4]. Furthermore, only a few studies confirming the feasibility of anatomic segmentectomy in clinical-T1c NSCLC have been conducted. Actually, several retrospective or population-based database studies demonstrated that the lobectomy still confers a superior survival advantage for T1c (>2–3 cm) NSCLC compared with segmentectomy [5–9].

Meanwhile, as shown in the several nationwide studies, the prognosis of early-stage NSCLC clearly differs based on the presence of ground-glass opacity (GGO) component on thin-section computed tomography (CT) scan [10–16]. Radiological part-solid lung adenocarcinoma demonstrates favourable prognosis (>90%), regardless of the size of the solid component in cases where the tumour showed a GGO component [13]. Theoretically, segmentectomy to preserve the lung parenchyma is expected to become the next novel surgical alternative to lobectomy for these oncologically indolent lesions, even when the tumour size is >2 cm [17]. In fact, segmentectomy in stage IA radiological solid-predominant (i.e. 0.5 < consolidation tumour ratio < 1.0) NSCLC of >2 cm as a maximum tumour size excluding pure-solid NSCLC resulted in favourable oncologic outcomes, which showed an acceptable loco-regional cancer control and excellent overall survival (OS) of >90% [17]. On the contrary, radiological pure-solid NSCLC demonstrated unfavorable biology and a significantly dismal prognosis due to its oncologically highly invasive nature compared with the part-solid type. To date, no study has investigated whether the anatomical segmentectomy is a promising surgical strategy for T1c radiological pure-solid NSCLC [10–16]. Based on these clinical backgrounds, the indication of segmentectomy for T1c radiological pure-solid NSCLC remains controversial as it is associated with insufficient lung cancer control.

Hence, the present study aimed to evaluate the outcomes of segmentectomy and lobectomy in T1cN0M0 pure-solid NSCLC, with a focus on the survival outcomes, causes of death or recurrence patterns between segmentectomy and lobectomy, using multivariable or propensity score-matched analyses.

## MATERIALS AND METHODS

### Ethics statement

The medical records of each patient were retrospectively reviewed, and the requirement for obtaining informed consent was waived by the Institutional Review Board of the Juntendo University School of Medicine, Tokyo, Japan (IRB number: E22-0401).

### Study population

Between 2008 and 2020, we retrospectively reviewed the data of 426 surgically resected clinical T1c (>2–3 cm) node-negative NSCLC patients with a radiological pure-solid appearance on a thin-section CT scan. At our institute, the 8th edition of the TNM Classification of Malignant Tumours were used for clinical staging [18]. None of the variables examined in this study had missing data. Patients whose disease stage was determined preoperatively by thin-section CT and complete resection and did not receive preoperative chemotherapy and/or radiotherapy were included in the study. Patients who underwent bi-lobectomy, sleeve lobectomy or pneumonectomy were excluded. With regard to the nodal status, clinical N0 indicated nonenlarged lymph nodes (short axis of <10 mm) on a thin-section CT scan. Invasive modalities for mediastinal lymph node staging, such as mediastinoscopy and endobronchial ultrasound-guided transbronchial needle aspiration, were performed preoperatively to confirm the node-negative status in patients who exhibited positive results on positron emission tomography examination.

### Radiological evaluation of thin-section CT scan findings

In all patients, the preoperative thin-section CT scan findings were reviewed in detail by the authors (A.H., T.M. and K.S.) and a radiology oncologist. Tumour size was determined preoperatively based on the thin-section CT findings with a maximum slice thickness of 2 mm collimation. The lung was photographed following appropriate window settings: window level of −500 to −700 H and window depth of 1000–2000 H as a lung window, and window level of 30–60 H and window depth of 350–600 H as a mediastinal window. The consolidation component was defined as an area of increased opacification that completely obscured the underlying vascular markings. GGO was defined as an area with a slight, homogenous increase in density that did not obscure the underlying vascular markings [13]. Furthermore, The consolidation tumour ratio was defined as the ratio of the maximum size of consolidation to the maximum tumour size on thin-section CT scan [19]. In this study, eligible tumours had radiologically pure-solid appearances without a GGO component on thin-section CT scan (i.e. consolidation tumour ratio = 1.0) [13].

### Operation policy

In the lobectomy arm, lobectomy with nodal dissection was performed. The resection of more than one lobe was not permitted. In the segmentectomy arm, anatomic segmentectomy with nodal dissection was performed by removing one or more pulmonary segments. The margin distance between a tumour and the intersegmental plane was checked based on thin-section CT scan in all 3 planes (i.e. axial, sagittal and coronal view), and a resection margin of >2 cm or equal to the tumour diameter by the CT imaging was confirmed preoperatively. Segmental fissures were essentially taken by the stapler. If a sufficient surgical margin could not be surgically ensured, the resection line was extended to the adjacent segment of the lung to ensure an adequate resection margin.

### Follow-up policy

Postoperative adjuvant chemotherapy with tegafur-uracil and cisplatin plus vinorelbine were recommended for patients with stage IB and stage II or IIIA disease, respectively [20, 21]. Measurement of tumour markers, chest X-ray and enhanced chest CT were performed at least every 6 months during the first 5 years and at least every 12 months thereafter. All of the data regarding the cause of death or the sites of recurrences were gathered from the medical record weekly. Furthermore, they have been confirmed annually in our department.

### Statistics

The categorical variables were reported as frequencies and percentages, while continuous variables were reported as median (interquartile range) or mean (standard deviation) as appropriate. For categorical variables, comparisons between groups were performed using chi-squared tests. Continuous variables were compared using the Wilcoxon rank sum test. Cox proportional hazards model was addressed to adjust the effect of segmentectomy by possible confounders, using SPSS Statistics 27 (IBM Inc., USA). Univariable and multivariable analyses were performed to adjust for the confounding variables affecting the survival. The preoperative comorbid status was evaluated using Charlson comorbidity index, and a score of ≥3 indicated a high comorbid status. The survival outcomes were estimated using the Kaplan–Meier method and compared using the log-rank test across study groups. The date of surgical resection was set as the starting point; moreover, the date of death, recurrence, or survival follow-up was set as the endpoint for the calculation of OS and recurrence-free survival (RFS). To assess the recurrence, time to event endpoints were analysed using competing risks analysis. The risk of recurrence was estimated using a cumulative incidence function, which accounted for death without recurrence as a competing event. Differences in cumulative incidence of recurrence between groups were assessed using the methods of Gray. The difference was considered statistically significant when the *P*-value was <0.05 in the multivariable models.

## RESULTS

Table 1 demonstrates the demographic and clinicopathological variables of the enrolled patients with c-T1cN0M0 radiological pure-solid NSCLC. The patients’ median age was 70 years, and 283 (66.4%) patients were men. The median tumour size was 25 mm, and the SUVmax was 8.1. Adenocarcinoma developed in 275 patients (64.6%) and squamous cell carcinoma in 131 (30.7%) patients. Pathologically, nodal metastasis was detected in 104 (24.4%) patients, lymphatic invasion in 125 (29.3%) patients and vascular invasion in 190 (44.6%) patients. Of the 426 eligible patients, lobectomy was performed in 381 patients and segmentectomy in 45 patients. The segmentectomy arm was older (*P* = 0.008), had male predominance (*P* = 0.088) and had higher Charlson comorbidity index (*P* = 0.001) compared with the lobectomy arm; meanwhile, no significant difference was observed in the maximum tumour size, SUVmax, frequency of nodal or lymphatic metastasis and vascular invasion between the 2 study arms. Table 2 shows the tumour locations and operative details of segmentectomy. To secure the sufficient surgical margin, segmentectomy with 3 or more resected segments was performed in 18 (40.0%) patients and S6 segmentectomy in 14 (31.1%) patients. There were no conversion cases from segmentectomy to lobectomy in the enrolled patients.

**Table 1:** Clinicopathological characteristics of T1c radiological pure-solid NSCLC based on the operative procedures

	Overall (*n* = 426)	Lobectomy (*n* = 381)	Segmentectomy (*n* = 45)	*P*-value^a^
Age (years), median (IQR)	70 (64–76)	69 (64–68)	74 (68–79)	0.008
Sex (male), *n* (%)	283 (66.4)	248 (65.1)	35 (77.8)	0.088
Side (right), *n* (%)	258 (60.6)	240 (63.0)	18 (40.0)	0.003
Pack-year smoking, median (IQR)	36.0 (1.0–60.0)	34.0 (0–56.0)	50.0 (13.5–70.5)	0.033
Charlson comorbidity index (high), *n* (%)	61 (14.3)	47 (12.2)	14 (31.1)	0.001
COPD, *n* (%)	196 (46.0)	170 (44.6)	26 (57.8)	0.094
Interstitial pneumonia, *n* (%)	56 (13.1)	50 (13.1)	6 (13.3)	0.969
Serum CEA level (ng/ml), median (IQR)	3.6 (2.4–5.8)	3.6 (2.4–5.7)	3.5 (2.6–6.1)	0.854
Maximum standardized uptake value, median (IQR)	8.1 (4.6–11.7)	8.1 (4.6–11.7)	7.7 (4.8–12.0)	0.666
Maximum tumour size (mm), median (IQR)	25.0 (23.0–28.0)	25.0 (23.0–28.0)	24.0 (22.0–27.0)	0.201
Nodal involvement, *n* (%)	104 (24.4)	93 (24.4)	11 (24.4)	0.996
p-N1/N2, *n* (%)	45 (10.6)/59 (13.8)	37 (9.7)/56 (14.7)	8 (17.8)/3 (6.7)	
Pathological-stage (stage I), *n* (%)	301 (70.7)	268 (70.3)	33 (73.3)	0.677
Histology (Ad/Sq/other NSCLCs), *n* (%)	275 (64.6)/131 (30.7)/20 (4.7)	253 (66.4)/111 (29.1)/17 (4.5)	22 (48.9)/20 (44.4)/3 (6.7)	0.067
Lymphatic invasion (yes), *n* (%)	125 (29.3)	115 (30.2)	10 (22.2)	0.267
Vascular invasion (yes), *n* (%)	190 (44.6)	172 (45.1)	18 (40.0)	0.511
Postoperative chemotherapy (yes), *n* (%)	132 (31.0)	123 (32.3)	9 (20.0)	0.092

a *P*-value in χ^2^ test, Student’s *t*-test or Wilcoxon rank sum test.

CEA, carcinoembryonic antigen; COPD, chronic obstructive pulmonary disease; IQR: interquartile range; NSCLC, non-small-cell lung cancer.

**Table 2:** Tumour locations and operative details of segmentectomy

	Lobectomy (*n* = 381)	Segmentectomy (*n* = 45)
Right upper lobe, *n* (%)	113 (29.7%)	6 (13.3%)
S1 segmentectomy		2
S2 segmentectomy		3
S1 + 2 segmentectomy		1
Right middle lobe, *n* (%)	29 (7.6%)	0 (0%)
Right lower lobe, *n* (%)	100 (26.2%)	12 (26.7%)
S6 segmentectomy		9
S8 segmentectomy		2
Basal segmentectomy		1
Left upper lobe, *n* (%)	79 (20.7%)	21 (46.7%)
S1 + 2 + 3 segmentectomy		16
S3 segmentectomy		1
Lingular segmentectomy		4
Left lower lobe, *n* (%)	60 (15.8%)	6 (13.3%)
S6 segmentectomy		5
S8 segmentectomy		1

**Figure 1:**
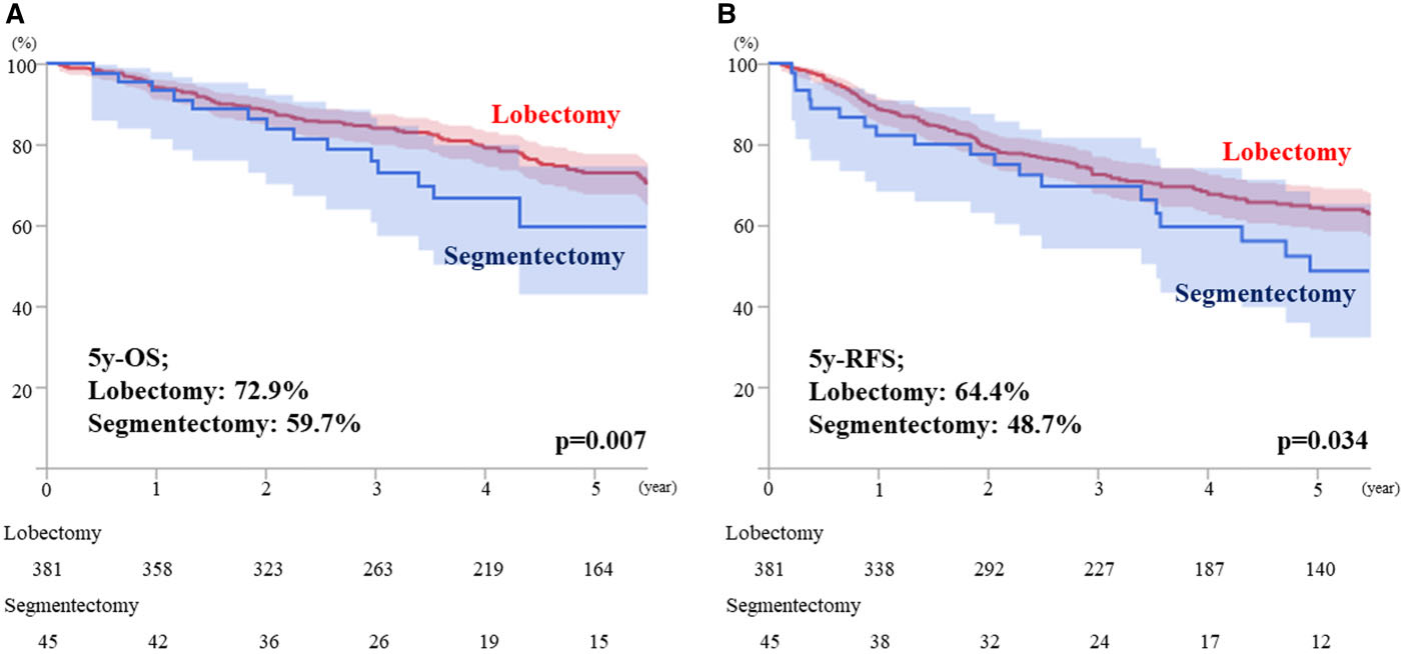
Both OS and RFS are shown based on the operative procedures performed in T1c radiologically pure-solid NSCLC patients. The survival outcomes of the lobectomy arm were significantly better compared with those of the segmentectomy arm (5-year OS: 72.9% vs 59.7%, *P* = 0.007; 5-year RFS: 64.4% vs 48.7%, *P* = 0.034), with a median follow-up time of 53.0 years. NSCLC: non-small-cell lung cancer; OS: overall survival; RFS: recurrence-free survival.

The result of the Cox proportional hazards regression analysis of OS is shown in Table 3. The multivariable analysis revealed that lobectomy was an independent prognosticator of better OS (hazard ratio 0.596, 95% confidence interval 0.366–0.969, *P* = 0.037). As shown in Fig. 1, the OS and RFS were significantly better in the lobectomy arm compared with that in the segmentectomy arm (5-year OS: 72.9% vs 59.7%, *P* = 0.007; 5-year RFS: 64.4% vs 48.7%, *P* = 0.034), with a median follow-up time of 53.0 months.

**Table 3:** Cox proportional hazard model for the overall survival

Variable	Univariable		Multivariable	
	HR (95% CI)	*P*-value^a^	HR (95% CI)	*P*-value^a^
Age	1.042 (1.021–1.064)	<0.001	1.043 (1.020–1.065)	<0.001
Sex (female)	0.538 (0.360–0.803)	0.002	0.582 (0.382–0.885)	0.011
Charlson comorbidity index (high)	2.622 (1.713–4.013)	<0.001	2.266 (1.446–3.551)	<0.001
Side (left)	1.172 (0.827–1.667)	0.373		
Pack-year smoking	1.002 (0.998–1.007)	0.300		
COPD (no)	1.151 (0.806–1.643)	0.439		
Interstitial pneumonia (no)	0.477 (0.307–0.742)	0.001	0.488 (0.305–0.782)	0.003
Serum CEA level (ng/ml)	1.016 (1.000–1.033)	0.050	1.011 (0.992–1.029)	0.256
Maximum standardized uptake value	0.987 (0.950–1.025)	0.488		
Maximum tumour size (mm)	1.062 (1.001–1.128)	0.047	1.045 (0.979–1.114)	0.187
Operative mode (lobectomy)	0.528 (0.330–0.843)	0.008	0.596 (0.366–0.969)	0.037
Histology (Adenocarcinoma)	0.720 (0.505–1.027)	0.070		
Pathological stage II or more	1.770 (1.242–2.522)	0.002	1.636 (1.130–2.369)	0.009
Lymphatic invasion (no)	0.546 (0.384–0.776)	0.001	0.441 (0.292–0.667)	<0.001
Vascular invasion (no)	0.629 (0.443–0.894)	0.010	0.824 (0.558–1.216)	0.329
Postoperative chemotherapy (no)	0.923 (0.645–1.319)	0.659		

a *P*-value in the Cox proportional hazard model.

CEA: carcinoembryonic antigen; CI: confidence interval; COPD: chronic obstructive pulmonary disease; HR: hazard ratio.

**Figure 2:**
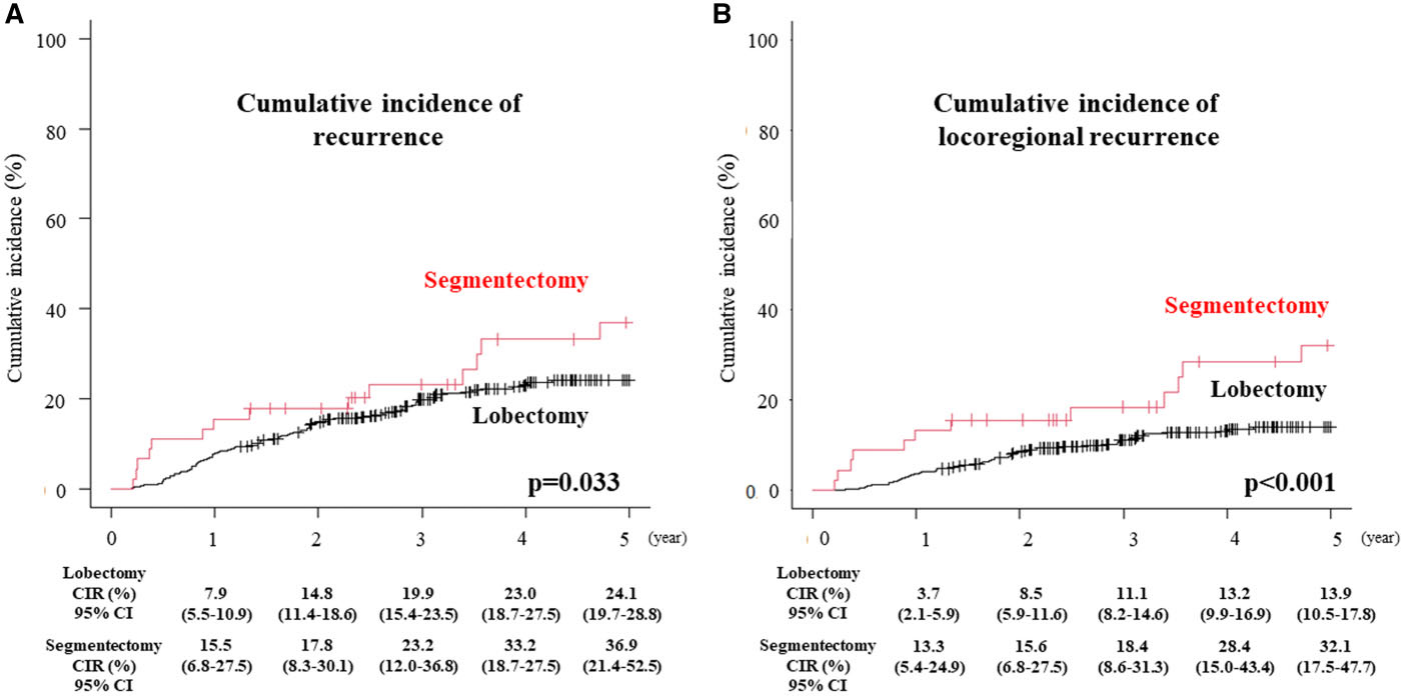
Cumulative incidence or recurrence (CIR) was shown based on the operative procedures performed in T1c radiologically pure-solid NSCLC patients. NSCLC: non-small-cell lung cancer.

Table 4 demonstrates the cause of death and recurrence patterns according to the type of surgery. Among them, the proportion of non-lung cancer-related death was similar between the 2 study arms (9.4% in the lobectomy group and 6.7% in the segmentectomy). By contrast, the proportion of lung cancer-related death was 1.9-fold higher in the segmentectomy arm (26.7%) compared with that in the lobectomy arm (14.2%). Furthermore, as shown in Fig. 2, the cumulative incidence or recurrence was higher in the segmentectomy arm compared to the lobectomy arm (5-year cumulative incidence or recurrence 36.9% vs 24.1%, *P* = 0.033). In particular, the cumulative incidence of loco-regional recurrence was significantly higher in the segmentectomy arm compared to the lobectomy arm (5-year cumulative incidence of loco-regional recurrence 32.1% vs 13.9%, *P* < 0.001). Meanwhile, no clear differences were observed in the cumulative incidence of distant recurrence between segmentectomy and lobectomy (15.6% vs 16.5%, *P* = 0.708). With regard to the sites of loco-regional recurrence (Table 4), the proportion of recurrence at the resected margin (6.7%), bronchial stump (4.4%), hilar or mediastinal node (15.5%) or ipsilateral lung (13.3%) in the segmentectomy arm was remarkable compared with that in the lobectomy arm.

**Table 4:** Cause of death and details of recurrence patterns

	Lobectomy (*n* = 381)	Segmentectomy (*n* = 45)
Cause of death at 5 years (%), *n* (%)		
Lung cancer	54 (14.2)	12 (26.7)
Other than lung cancer	36 (9.4)	3 (6.7)
Recurrent patterns, *n* (%)		
Locoregional only	24 (6.3)	8 (17.8)
Locoregional + distant	27 (7.1)	6 (13.3)
Distant only	41 (10.8)	3 (6.7)
Sites of loco-regional recurrence, *n* (%)		
Resected margin of lung parenchyma	0 (0)	3 (6.7)
Bronchial stump	4 (1.0)	2 (4.4)
Mediastinal lymph node	28 (7.3)	5 (11.1)
Hilar lymph node	6 (1.6)	2 (4.4)
Ipsilateral lung	5 (1.3)	6 (13.3)
Pleural effusion	8 (2.1)	0 (0)
Pleural dissemination	6 (1.6)	1 (2.2)
Intrapericardial effusion	1 (0.3)	0 (0)
Sites of distant recurrence, *n* (%)		
Brain	17 (4.5)	4 (8.9)
Liver	3 (0.8)	0 (0)
Bone	29 (7.6)	3 (6.7)
Adrenal	3 (0.8)	1 (2.2)
Contralateral lung	30 (7.9)	4 (8.9)
Subclavian lymph node	2 (0.5)	1 (2.2)
Other sites	3 (0.8)	1 (2.2)

## DISCUSSION

In the era where several phase III trials demonstrated the equivalent or superior survival outcomes of segmentectomy for small-sized NSCLC [1–3], whether anatomical segmentectomy can be indicated for T1cN0M0 NSCLCs while minimizing local recurrence and maximizing survival outcomes needs to be elucidated further. However, T1c radiological pure-solid NSCLC is highly invasive [10–16], and no previous studies have investigated whether the anatomical segmentectomy is a promising surgical strategy for T1cN0M0 radiological pure-solid NSCLC. In the present study, Cox proportional hazards model elucidated that the anatomical segmentectomy was significantly associated with worse survival outcomes through a multivariable analysis. Furthermore, the OS and RFS of the segmentectomy arm were significantly inferior to those of the lobectomy arm.

Although the data of lung cancer recurrence or cause of death were not performed multivariate adjustment, the main reason of the worse survival outcomes of segmentectomy would be higher frequency of loco-regional cancer recurrence and the subsequent lung cancer-related death. In this study, ipsilateral lung or lymph nodal recurrence were more frequent than lung staple line recurrences. Insufficiency of nodal dissection or loco-regional cancer control might cause cancer recurrence after segmentectomy in early phase. Furthermore, loco-regional recurrence requires additional treatment including chemotherapy, radiotherapy or surgery, which give the additional physiological burden to the patients [1]. In addition, repeated surgery for the loco-regional failures is technically demanding [22]. Furthermore, loco-regional recurrence as a first detected metastatic site would attribute to the following systemic metastatic disease. Because T1c pure-solid lung cancer shows oncologically high malignant features, our result would indicate the significance of strict loco-regional cancer control.

Meanwhile, we have to emphasize the fact that this study includes ‘lobe-like’ segmentectomies—i.e. a left upper division or lingulectomy is arguably similar to a RUL or RML. It is considered that a ‘lobe-like’ segmentectomy has very different oncologic and physiologic implications than a single segment. Despite that, loco-regional cancer control is inferior in the segmentectomy arm. This aspect only underscores even more the importance of finding worse outcomes after segmentectomy. To the best of our knowledge, this study is the first to report the oncological outcomes, cause of death, or recurrent patterns of clinical T1c radiologically pure-solid NSCLC patients who underwent segmentectomy and lobectomy. Further discussion is warranted with regard to this topic to properly apply the surgical procedures for the T1c pure-solid NSCLC.

Since the conduct of the Lung Cancer Study Group (LCSG) trial in 1995, lobectomy has been considered as a gold standard treatment for peripherally located T1N0 NSCLC (3 cm or less in size) [4]. Furthermore, several previous studies using the population-based database demonstrated that lobectomy still confers a significant survival benefit compared with segmentectomy for stage IA NSCLC of >2 cm in tumour size [5, 6]. Notably, these studies reported a 5-year OS of 60% [5, 6]. A clear radiological definition was not provided in these previous studies; however, it is speculated that the previously enrolled T1c NSCLC patients [5, 6] more likely show a pure-solid appearance without a GGO component on radiological examination, when referring to the survival data of stage IA NSCLC patients with a GGO component on thin-section CT scan [13]. Recently, the concept of ‘less is more’ is considered to be an important notion as the next surgical strategy for small-sized NSCLC [1]. One important lesson for general thoracic surgeons is that a less invasive procedure can possibly provide long-term survival benefits for patients with early-stage indolent lung cancer, reducing the physiological burden and lung cancer recurrence. Hence, patients with limited surgical indications refer those with a GGO component on radiological examination. On the contrary, the result of LCSG trial [4], several retrospective studies [5–9], and our present study strongly support that adequate loco-regional cancer control is mandatory to achieve the long-term survival outcomes for patients with T1c (>2–3 cm) radiological pure-solid, i.e. highly invasive, NSCLC. Further studies are required to address this clinical issue, however, we should be cautious to the surgical indication of segmentectomy for T1c radiological pure-solid NSCLC, which may cause the avoidable lung cancer recurrence.

### Limitations

There are some limitations to this study. First, this study was based on a retrospective and single-institution Japanese database. Second, it was subject to the inherent biases of a retrospective study, the most important of which is the selection bias in the allocation of treatment. Due to the retrospective nature, it seems that segmentectomy arm was relatively small number, and preferably performed among those patients with poorer profile. Therefore, it might be hypothesized that observed differences could be partially explained by such baseline differences instead. Therefore, these factors should be taken into account to interpret the current data. Third, the data of margin distance based on the surgical specimen was not presented for all in this study. However, there is increasing interest in this area in which treatment for early-stage NSCLC is being developed. Therefore, this analysis has immense clinical implications to our daily practice.

## CONCLUSION

In conclusion, anatomic segmentectomy was significantly associated with worse survival and insufficient loco-regional cancer control in the setting of T1cN0M0 radiological pure-solid NSCLC. To date, lobectomy is the standard surgical mode, and intentional segmentectomy should be applied with great caution for T1c radiological pure-solid NSCLC on thin-section CT scan.

## Data Availability

The data underlying this article cannot be shared publicly due to the privacy of individuals that participated in the study.

## ABBREVIATIONS

CT: Computed tomography
GGO: Ground-glass opacity
NSCLC: Non-small-cell lung cancer
OS: Overall survival
RFS: Recurrence-free survival
